# A multi-label classification model for full slice brain computerised tomography image

**DOI:** 10.1186/s12859-020-3503-0

**Published:** 2020-11-18

**Authors:** Jianqiang Li, Guanghui Fu, Yueda Chen, Pengzhi Li, Bo Liu, Yan Pei, Hui Feng

**Affiliations:** 1 School of Software Engineering, Beijing University of Technology, Beijing, 100124 China; 2 Beijing Engineering Research Center for IoT Software and Systems, Beijing, 100124 China; 3 Department of Neurosurgery, Tianjin Huanhu Hospital, Tianjin, 300350 China; 4 Computer Science Division, University of Aizu, Aizuwakamatsu, 965-8580 Japan

**Keywords:** Bioinformatics, Brain computerised tomography, Machine learning, Deep learning, Computer aided diagnosis

## Abstract

**Background:**

Screening of the brain computerised tomography (CT) images is a primary method currently used for initial detection of patients with brain trauma or other conditions. In recent years, deep learning technique has shown remarkable advantages in the clinical practice. Researchers have attempted to use deep learning methods to detect brain diseases from CT images. Methods often used to detect diseases choose images with visible lesions from full-slice brain CT scans, which need to be labelled by doctors. This is an inaccurate method because doctors detect brain disease from a full sequence scan of CT images and one patient may have multiple concurrent conditions in practice. The method cannot take into account the dependencies between the slices and the causal relationships among various brain diseases. Moreover, labelling images slice by slice spends much time and expense. Detecting multiple diseases from full slice brain CT images is, therefore, an important research subject with practical implications.

**Results:**

In this paper, we propose a model called the slice dependencies learning model (SDLM). It learns image features from a series of variable length brain CT images and slice dependencies between different slices in a set of images to predict abnormalities. The model is necessary to only label the disease reflected in the full-slice brain scan. We use the CQ500 dataset to evaluate our proposed model, which contains 1194 full sets of CT scans from a total of 491 subjects. Each set of data from one subject contains scans with one to eight different slice thicknesses and various diseases that are captured in a range of 30 to 396 slices in a set. The evaluation results present that the precision is 67.57%, the recall is 61.04%, the F1 score is 0.6412, and the areas under the receiver operating characteristic curves (AUCs) is 0.8934.

**Conclusion:**

The proposed model is a new architecture that uses a full-slice brain CT scan for multi-label classification, unlike the traditional methods which only classify the brain images at the slice level. It has great potential for application to multi-label detection problems, especially with regard to the brain CT images.

## Background

Brain computerised tomography (CT) scans are the most commonly used diagnostic tools for patients with brain trauma or brain diseases, such as brain tumour, intracranial haemorrhage, calvarial fracture, and other injuries. Brain CT is also the first choice of imaging tool to learn the brain structure, and CT device is a common service available in most hospitals. CT scans present the great quality of visual information on internal organs. The advantage of using brain CT scans lies on its low cost and time-saving, so it is a great tool for preliminary diagnoses. As the brain CT scans have become widely available, doctors spend a considerable amount of time in reading and interpreting the results. Such delays in the diagnostic process can worsen injuries and cause unnecessary harm to the patients. Many researchers are therefore trying to use computer technologies to aid in diagnoses and treatments.

Nowadays, artificial intelligence techniques are developing rapidly. Deep learning models can learn a hierarchy of features by building high-level attributes from low-level ones. Utilising deep learning methods with medical knowledge is a valuable direction of study and presents an extraordinary potential for auxiliary medical treatment. Rajpurkar’s team has proposed an algorithm to detect the presence of 14 different pathologies from chest radiographs [1]. Jeffrey’s team developed an architecture that can be used for more than 50 common diagnoses using optical coherence tomography (OCT) [2]. These study works show that medicine as a field can benefit from deep learning method. Some algorithms can detect brain diseases. Gao’s team aimed to provide a method for early diagnosis of Alzheimer’s disease (AD) [3]. They used CT sets and clustered them into three groups as AD, lesions (e.g., tumour), and normal. They manually chose some images that contained visual lesion features (e.g., tumour) from full-slice brain CT as the experimental data. Their method used both two-dimension (2-D) and 3-D images for evaluation. In reference [4], the investigators utilised 904 cases containing 14,758 brain CT images to obtain excellent performance on their retrospective and prospective dataset.

Most methods select samples from a set of brain CT images for classification and this requires labelling the images one by one. More importantly, if we consider the images in a set of brain CT scans separately, the dependence information between each slice is missing. In clinical diagnoses, a doctor provides a final diagnosis conclusion by observing a full sequence of brain CT images. A patient may have multiple brain diseases concurrently, and these brain diseases may have a causal relationship. Certain diseases can cause other pathological and structural changes in the brain, which can further cause or precipitate other diseases. However, traditional multi-class classifications cannot take into account the relationships between diseases. They consider each disease category separately, which can not apply to patients with multiple brain diseases, as commonly encountered in clinical cases. Current researches on brain CT for brain disease detection mainly focus on a single image slice, i.e., single-image-based detection, where the target images are selected from a full set of slices by a clinical expert. In practice, the doctor generally detects diseases by quickly browsing the full set of slices and selecting one or several images for detailed checks to make a decision. Our research philosophy is motivated from this observation, i.e., we attempt to directly use a full set of slices for detection of brain diseases or conditions, where the single-image-based features and slice dependencies are combined to build a multi-label prediction model. It presents one of the original contributions in this work.

**Fig. 1:**
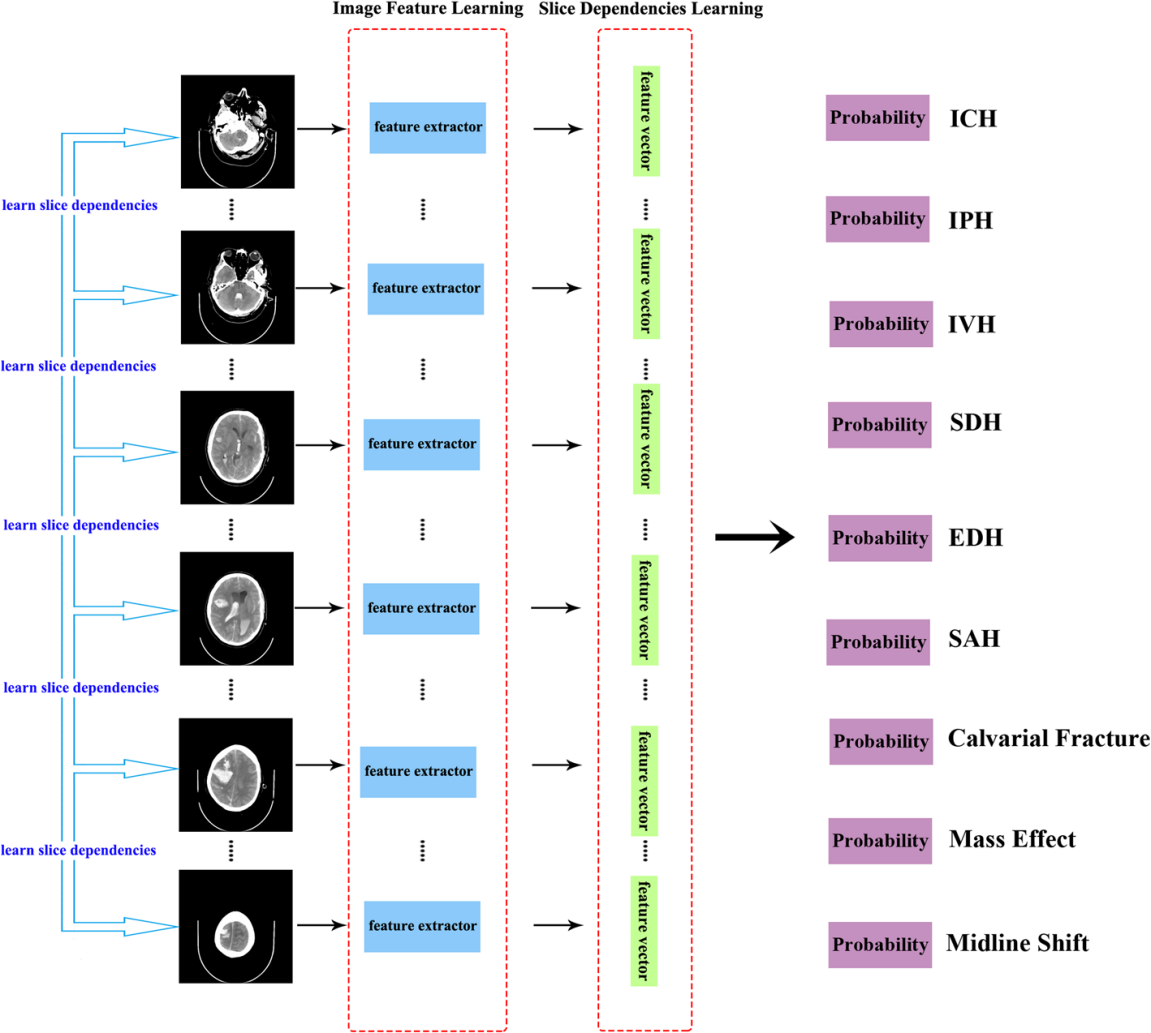
Example of the proposed model. We propose an architecture that utilizes a convolutional neural network (CNN; VGG16) to learn image features from a variable-length series of brain CT scans and that uses a recurrent neural network (RNN; GRU) to learn slice dependencies for predicting nine categories of brain CT images simultaneously. The nine categories of brain CT images are denoted as intracranial haemorrhage, intraparenchymal, intraventricular, subdural, extradural, subarachnoid, calvarial fracture, mass effect, and midline shift

In this paper, we propose a model called the slice dependencies learning model (SDLM) that composes image feature learning and slice dependencies learning for multi-label classifications from variable-length series of brain CT images. Our model can effectively learn image features and slice dependencies in an end-to-end manner and only requires labelling of a full set of CT images. This is a very convenient and time-saving method when labelling training data. The image feature learning part learns the full-slice brain CT images using a convolutional neural network (CNN) called VGG [5], which is pre-trained on ImageNet [6]. The feature learning model learns the features over classes *P*(*y*|*x*_1_,*x*_2_,...,*x*_*t*_) given a sequence of inputs *x*_1_,*x*_2_,...,*x*_*t*_ together, rather than a single input *x*. We use a recurrent neural network (RNN) called the gated recurrent unit (GRU) [7] in the slice dependencies learning part. The slice dependencies between the variable length slices and the causal relationship among multiple diseases are obtained from RNN. We conducted experimental evaluations with different CNN and RNN settings to select a better model configuration. The experiments present that the VGG16 and GRU perform better than other pairs in our proposed method. The architecture of this model is shown in Fig. 1.

In the following sections, we first review the related works on brain CT classifications and multi-label image classification areas in the section of related works and techniques. In the section of methods, we introduce the methods of image feature learning and slice dependencies learning to design and construct our model and method architecture. Section of experimental evaluations presents experiments with datasets, training details, and the experiments between different CNN and RNN pairs. We also make a comparative study on our model with the 3-D model. Section of results presents the primary discoveries of our work. In section of discussion, we discuss the evaluation results of our proposal and explain how to assist medical diagnosis using our proposal. In section of conclusion, we conclude our work and present some open topics and subjects for future work.

## Related works and techniques

Our work is in the field of brain CT classification, especially the study of multi-label classification problems. In this section, we briefly review some related works in two subjects, i.e., the brain image classification and the multi-label image classification.

### Brain image classification

**Fig. 2:**
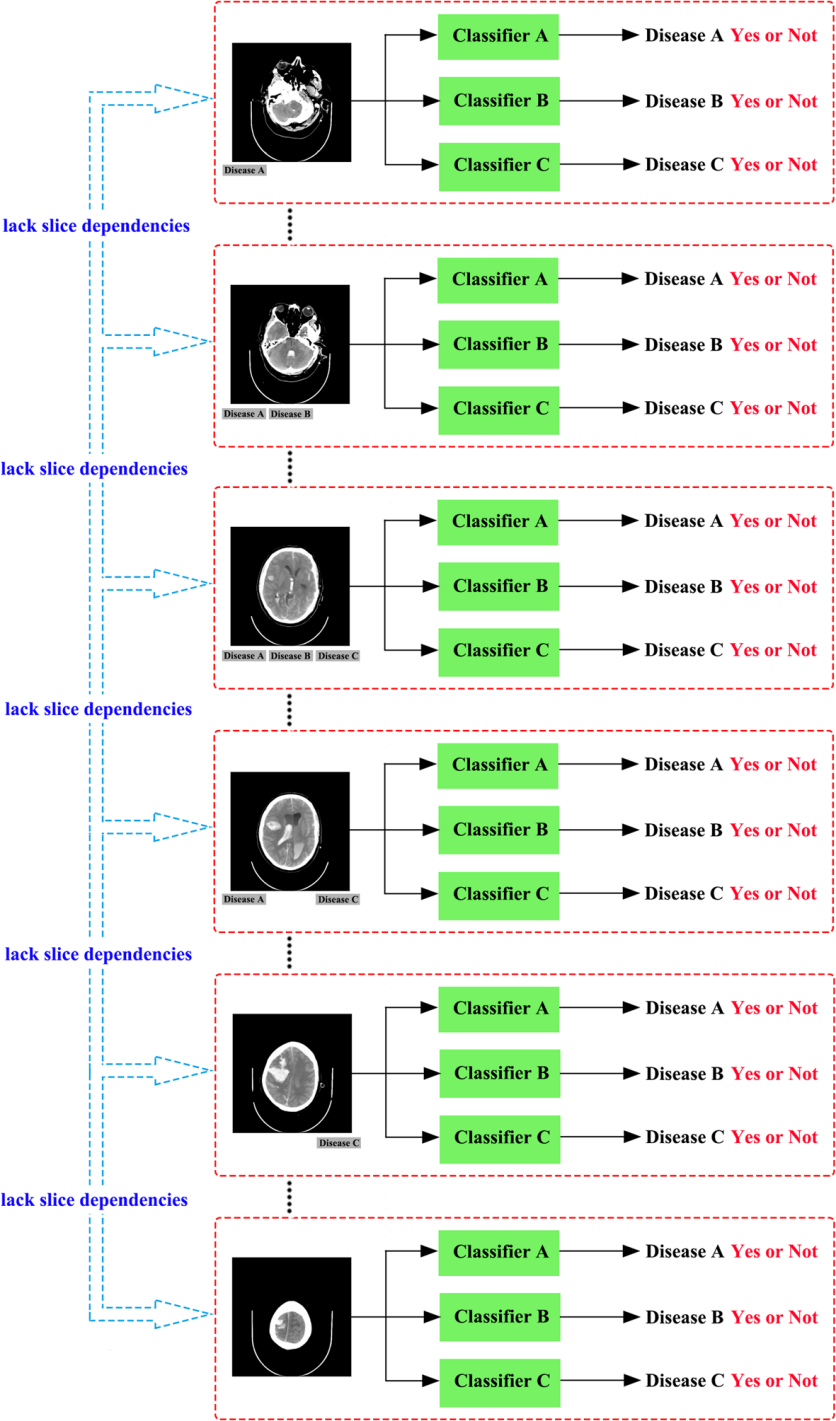
A classical architecture for training *n* classifier for *n* diseases in a single slice of brain CT scan, which ignores slice dependencies

Brain CT classification using machine learning methods has great potential for the early detection of brain injuries. In reference [8], the authors utilised wavelet energy to extract features and used support vector machine (SVM) as a classifier to diagnose the magnetic resonance (MR) brain images as normal or abnormal. The dataset consisted of 90 T2-weighted MR brain images. The method obtained a high accuracy, but the images were only clustered into two categories, i.e., normal and abnormal, which is in line with many other kinds of research such as in references [9–11]. In reference [3], the investigators aimed to provide supplementary information for early diagnosis of Alzheimer’s disease (AD). This is a state-of-the-art method that uses both 2-D and 3-D images in their study. Many research works, such as references [8–11], detect brain diseases from a single image slice and train *n* classifiers for multi-class classification (*n* represents the categories of the various diseases). An example of these methods is shown in Fig. 2. These works achieved high accuracies, but they only used a single brain CT image as training and predict sample. Brain CT images are different from other medical images. They are a sequence of data consisting of a set of images, and often, slice dependencies exist between the different slices.

Some researches attempt to utilize a 3-D model to consider the slice dependencies in a full-slice scan. In the work of [12], they use 3-D MRI brain scans to classify Alzheimer’s disease versus mild cognitive impairment and normal controls. It is a good attempt to consider full-slice images in the brain scan. In the work of [13], they propose a fully automated deep learning framework which learns to detect brain haemorrhage using cross-sectional CT images. They created an ensemble of three different 3-D CNN architectures to improve the classification accuracy. The AUC of the ensemble of three architectures was 0.87. In work of [14], they assemble the largest dataset ever used for training a deep 3-D CNN to classify brain images as healthy, mild cognitive impairment (MCI) or Alzheimer’s disease (AD). This model does not need for elaborate feature engineering and the workflow is considerably simpler, which increases clinical applicability. In work of [15], they proposed a new multi-modal 3-D CNN model for classifying the Alzheimer’s disease. They used structural MR and FDG-PET images to capture the rich features of 3-D MR images and FDG-PET images. The four references ([12–15]), all use 3-D model to classify brain diseases. But, there is still a big challenge if a set of brain scan has multiply diseases together.

Disease diagnosis from medical images requires high quality and accuracy, which is a time-consuming task even for well-trained and experienced doctors. To assist this process, deep learning-based computational methods are desired but depend on a large number of labelled data. Labelling data takes a lot of time and consumes a large amount of manpower. In reference [4], 904 sets of data containing 14,758 images were classified into five ICH subtypes from brain CT scans. Five neuroradiology specialists with 9 to 34 years of experience labelled the dataset. Besides the time spent by these doctors, a more important issue is that in practice, a doctor cannot pick a single image from a full-slice brain CT scan to diagnose diseases. Indeed, a doctor observes all full-slice scans and observes on changes in brain structure based on the whole sequence of images to conclude the final diagnosis result. The model we proposed can perform multi-label classification on the premise of considering full-slice brain images. The model can consider the dependencies between slices and the causal relationships between diseases. Our model that only requires labels of full-slice brain CT scans, it can also save a lot of time in diagnosis.

### Multi-label image classification

A set of brain CT scans can be used to detect many diseases at the same time, so we treat this problem as a multi-label classification task. Deep CNNs have shown great success in the single-label image classification, but in the real world problems, images contain multiple labels corresponding to different objects in one image. Multi-label classification is, therefore, a crucial study subject in practice.

Conventional methods learn many binary classifiers for each label category and employ ranking or thresholds on the final classification results for multi-label classification tasks. This method works well but does not exploit the relationship between multiple labels. They cannot handle label co-occurrence dependencies if the multiple labels are dependent. The dependencies between labels are important because of the causal relationships between brain diseases. For example, brain tumours may oppress the brain structure, causing a midline shift. Consequently, we need to consider different brain diseases simultaneously.

In reference [16], RNNs were combined with CNNs to solve this problem. This method obtained success in single image multi-label classification and achieved better performance than the state-of-the-art multi-label classification models. In reference [17], a new method called hypotheses-CNN-pooling (HCP) was proposed, where an arbitrary number of object segment hypotheses were taken as the inputs, a shared CNN connected with each hypothesis. Finally, the CNN results from different hypotheses were aggregated with maximum pooling to produce the final multi-label predictions. This method also performed well in single-image multi-label classifications. These methods have obtained good performance in the multi-label classifications of single images, but brain CT is a sequence of images. We cannot consider it as a multi-label classification problem for a single image. In brain CT classification, we investigate the full series of brain CT scans for the multi-label classification problem.

## Methods

**Fig. 3:**
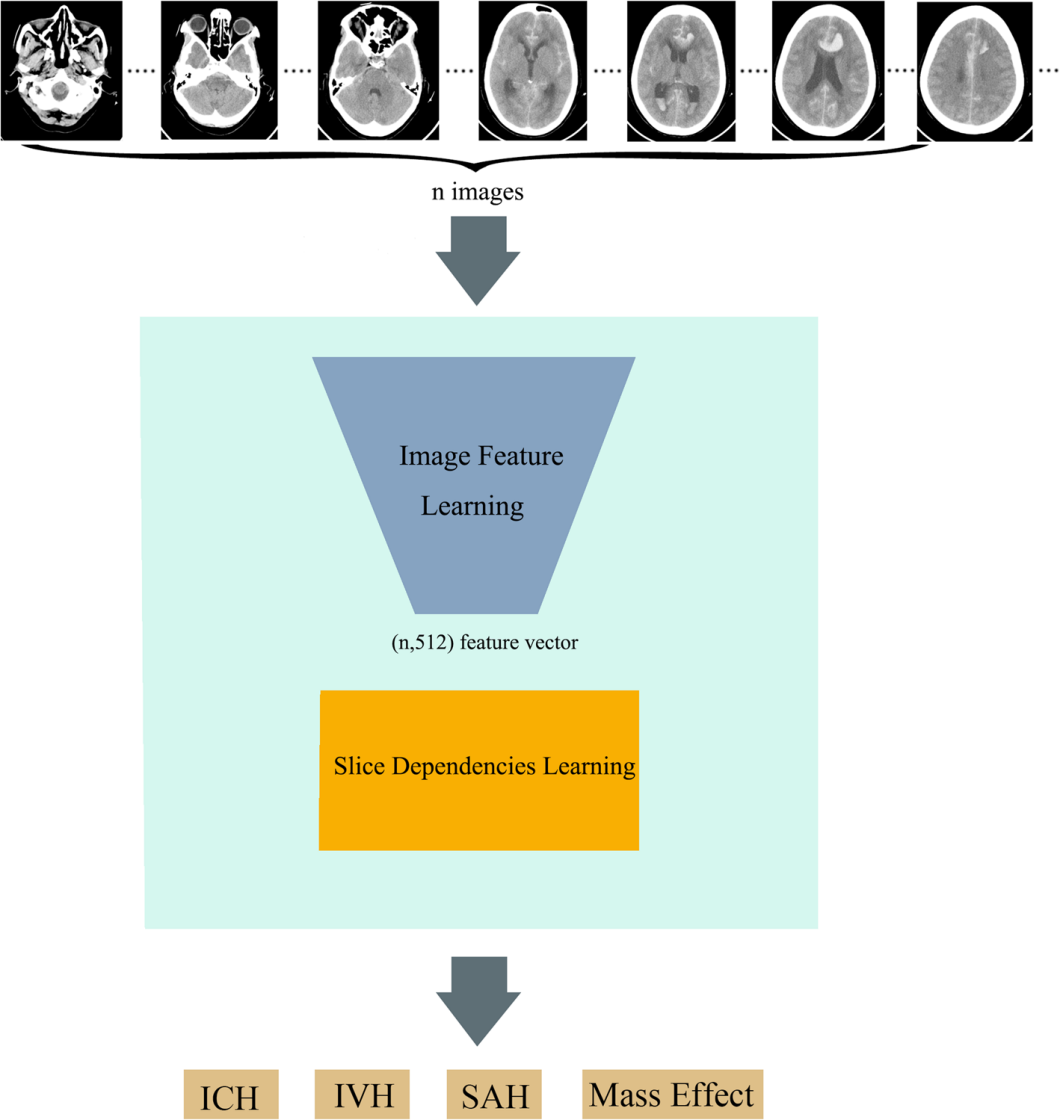
Architecture to predict multiple-labels from a set of brain CT scan

We propose a model called the SDLM for full-slice brain CT multi-label classification, which effectively learns both the image features and slice dependencies in an end-to-end manner. It is a sequence to sequence (seq2seq) model that can classify brain diseases into multiple categories at the same time. This model includes two parts: (1) image feature learning part, and (2) slice dependencies learning part. The first part learns the feature from a full set brain CT image automatically. The second one is employed to learn the dependencies among multiple slices, which provides the potential to handle the causal relationship between brain diseases. For the implementation of the proposed model, CNN is used for image feature learning, and RNN is used for the slice dependencies learning. The model for this method is presented in Fig. 3.

### Image feature learning and extraction

In the past century, to learn the intrinsic structure of data, many data representation learning methods have been proposed [18]. Some conventional methods, such as principal component analysis (PCA) [19], linear discriminant analysis (LDA) [20] and linear principal component discriminant analysis [21], have been proposed and widely used. In 2006, Hinton proposed the concept of deep learning, and this method successfully applied deep neural networks to dimensionality reduction. With the development of graphics processing units (GPU), deep learning has shown enormous advantages and has been employed in many areas, such as image recognition and speech recognition, etc. Deep learning uses many layers of linear or nonlinear processing units for feature extraction and transformation. It can learn multiple levels of features or representations of the data. Higher-level features are derived from lower level features to form a hierarchical representation.

CNNs are the neural networks that are mostly applied to vision recognition tasks. A CNN consists the convolutional layers, the pooling layers, the fully connected layers, and the normalization layers. The advantage of CNN lies on that it can make the forward function more efficient to implement and vastly reduce the number of parameters in the network. Parameter sharing schemes are used in the convolutional layers to reduce the number of parameters.

VGG Network is one of the CNN architectures designed in 2014 [5]. There are two kinds of VGG networks, VGG16 and VGG19, which have 16 and 19 layers, respectively. It is a famous model that achieves 92.7% top-5 test accuracy in ImageNet.

The residual neural network (ResNet) is an elegant architecture with skip connections that can be used to build a deeper neural network and to solve the degradation problem [22]. ResNet was used to train a neural network with 152 layers and still had a lower complexity than the VGG network.

The densely connected convolutional network (DenseNet) is a convolution neural network with dense connections [23]. It has a simple connectivity pattern to ensure maximum information flow between layers in the network. It connects all layers (with matching feature-map sizes) directly with each other.

**Fig. 4:**
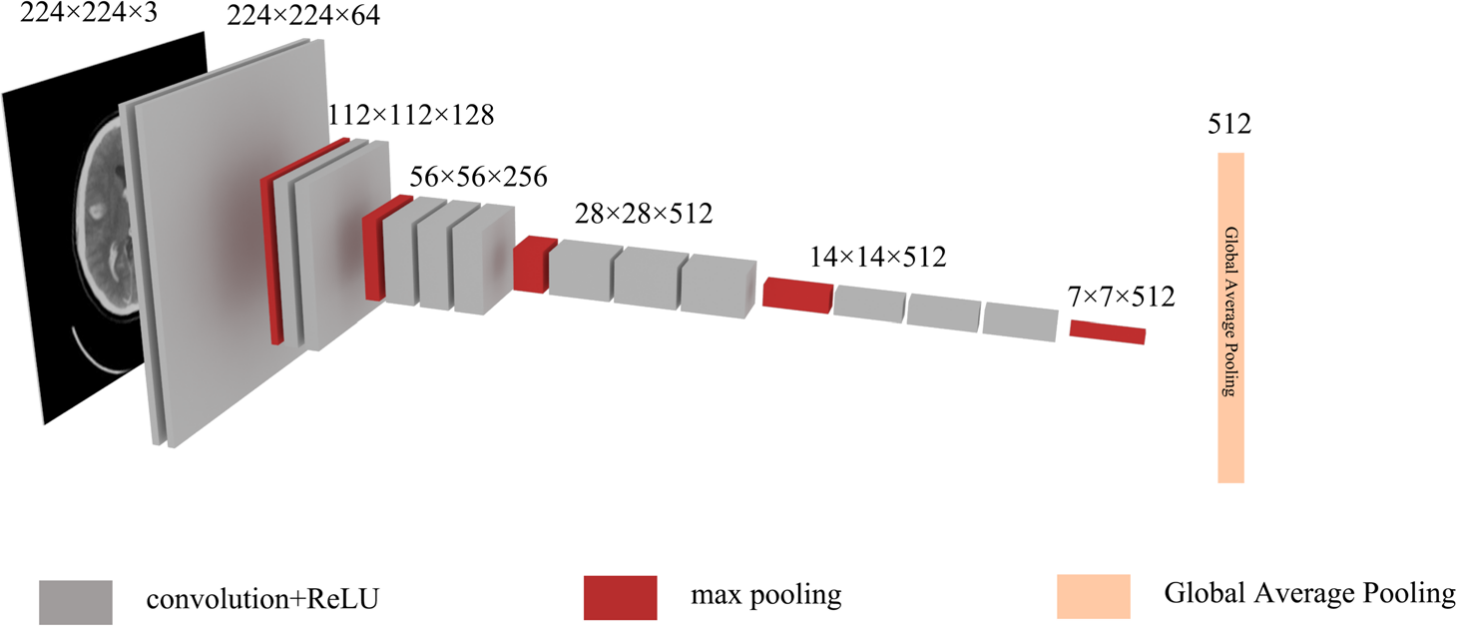
Feature learning model, VGG16, that can automatically learn features of a slice of brain images through convolution and pooling layers. Finally, through a global average pooling layer, we can obtain a vector that contains 512 dimensions

The pre-trained VGG, DenseNet, and ResNet models using the ImageNet [6] dataset are utilized in the comparison study. The VGG (DenseNet and ResNet) is implemented in 2-D. For each image from a set of brain CT, the feature learning model VGG (DenseNet and ResNet) generates a 512 (1024 and 2048) dimension vector. The schematic diagram of the learning process of VGG16 is illustrated in Fig. 4. For the *n* images in a full set of slices of brain CT, the VGG (DenseNet and ResNet) model learns and generates a *n*×512 (*n*×1024 and *n*×2048) feature matrix.

### Slice dependencies learning

Causal relationships exist among the nine categories of brain diseases studied in our work. Therefore, it is crucial to learn slice dependencies between the different slices. Learning only on a single slice is not recommended. The recurrent neural network (RNN) is a connectionist model that captures the dynamics of sequences by cycles in the network of nodes [24]. It is one of the neural networks that exhibit an outstanding performance using sequential learning. Consequently, the RNN is suitable for machine translation, speech recognition, etc. Conventional RNNs can learn complex temporal dynamics by mapping input sequences to a sequence of hidden states, and hidden states to outputs via the following recurrence equations (Eq. (1)):

1 $$  \begin{aligned} h_{t}&=\sigma(W_{hx} x_{t} + W_{hh} h_{t-1} + b_{h}). \\ y_{t}&=\sigma(W_{hy} h_{t} + b_{y}). \end{aligned}  $$

In Eq. (1), *x*_*t*_ is the input at time step *t*. *W*_*hx*_ is the matrix of convolutional weights between the input and the hidden layer and *W*_*hh*_ is the matrix of recurrent weights between the hidden layer and itself at adjacent time steps. At the time *t*, nodes with recurrent edges receive input from the current data point *x*_*t*_ and also from the hidden node *h*_*t*−1_, which is from the previous states. The output *y*_*t*_ at time *t* is calculated by the hidden node *h*_*t*_. *σ* is an activation function, such as a sigmoid function, rectified linear unit (ReLU). The vectors *b*_*h*_ and *b*_*y*_ are the bias parameters. Because of these, the RNN can learn the weights depending on current and past states.

Gated recurrent units (GRUs) [25] are a standard mechanism in recurrent neural networks. The GRU can be considered as a variation on the long short-term memory (LSTM) [26]. The gating mechanism is efficient, and this method can save many parameters than LSTM, but its performance was found to be similar to that LSTM in some applications.

2 $$  \begin{aligned} z_{t}&=\sigma_{g} (W_{z} x_{t} + U_{z} h_{t-1} + b_{z}).\\ r_{t}&=\sigma_{g} (W_{r} x_{t} + U_{r} h_{t-1} + b_{r}).\\ h'_{t}&=\sigma_{h} (W_{h} x_{t} + U_{h} (r_{t} \odot h_{t-1})+b_{h}).\\ h_{t}&= (1-z_{t}) \odot h_{t-1} + z_{t} \odot h'_{t}.\\ \end{aligned}  $$

In Eq. (2), *z*_*t*_ and *r*_*t*_ are the update gate and the reset gate at time step *t*, respectively. *h*_*t*_ is the output vector. These two vectors *z*_*t*_ and *r*_*t*_ decide what information should be passed to the output *h*_*t*_. *r*_*t*_ is the reset gate, which is used to determine the amount of past information that needs to be forgotten. *z*_*t*_ is the gate that determines whether to update the information or not. $h^{\prime }_{t}$ is the current memory content that uses the reset gate to store information from past states. *W* is the weight and *b* is the bias of each gate. *U* is the weight of the output h. *σ*_*g*_ is the sigmoid activation function and *σ*_*h*_ is the tanh function. ⊙ is the element-wise product.

The GRU has only two gates, i.e., the update gate and reset gate. The GRU will save lots of parameters compare with LSTM. Many experiments have shown that their performance is not considerably different. In this study, we compare the two RNN units and use the GRU as our sequence learning model.

### Model implementation, training and utilization

We use the mean squared error(MSE) as our loss function. We calculated the MSE using the following standard equation (Eq. (3)).

3 $$  MSE={\frac{1}{n}}{\sum_{i=1}^{n} (y_{i} - \hat{y_{i}})^{2}}.  $$

**Fig. 5:**
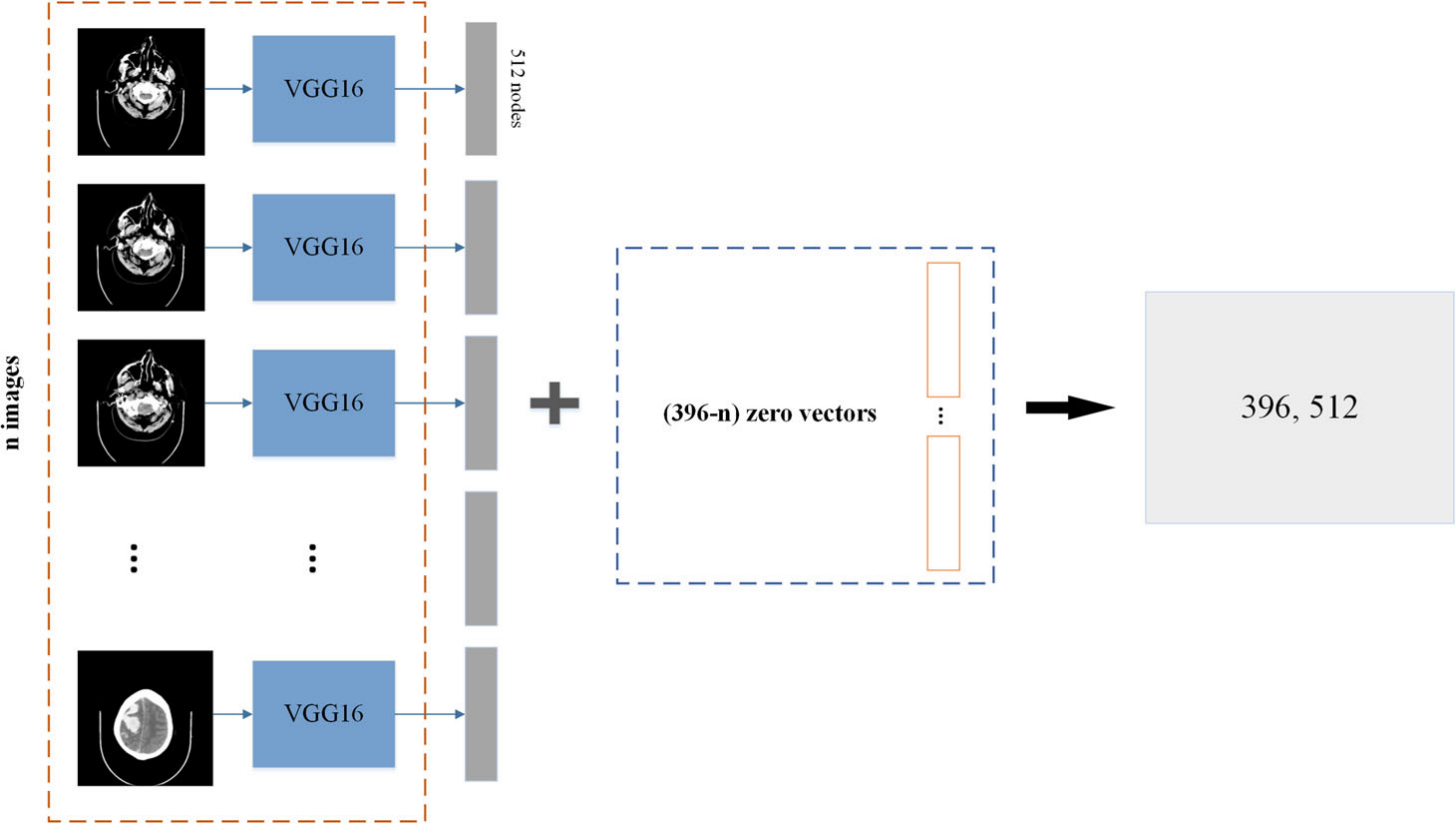
A set of data contain 396 images, for training reason we put the feature matrix into (396, 512) shape. We add (396-n) zero vectors to generate a (396, 512) matrix. Because of the masking layer, the zero vector will be filtered, and it will not affect training

In Eq. (3), *n* is the number of test samples, *y*_*i*_ is the true label, and $\hat {y_{i}}$ is the predicted label. Because each CT set is different, the number of slices depends on the thickness of the CT scan; however, no more than 396 images are used per set. So, we reshape the image sets into 396. If the set is less than 396 images, it will be filled by zeros and padded to 396 images. An example can be seen in Fig. 5.

We use the VGG16 model with weights pre-trained on the ImageNet classification challenge dataset to extract features of full-slice brain CT images. These weights are imported from the ones released by the VGG at Oxford. After learning through the 2-D convolutional neural network, the vector size of a CT image is 512. If the sequence CT scan has *n* images, the full-slice of the brain CT will be a matrix of (n ×512) dimensions. We use batch normalization [27] as our normalization function. The embedding of the GRU layers is 512, and dropout [28] is used to avoid over-fitting. We set the dropout rate to 0.2.

We add two fully connected layers after the GRU layer and use the ReLU as an activation function. We add dropout between each layer, the first and the second dropout rates are both 0.5. The batch size is set as 128, and 23 epochs are trained. The optimizer used is Adam [29]. Adam is an effective variant of an optimization algorithm called the stochastic gradient descent, which iteratively applies updates to parameters in order to minimize the loss during training and to avoid the gradient vanishing/exploding problems. The last layer contains *n* neurons to decode the probability of *n* diseases, and the activation function is a sigmoid function.

## Experimental evaluations

### Brain CT image data in evaluation

**Fig. 6:**
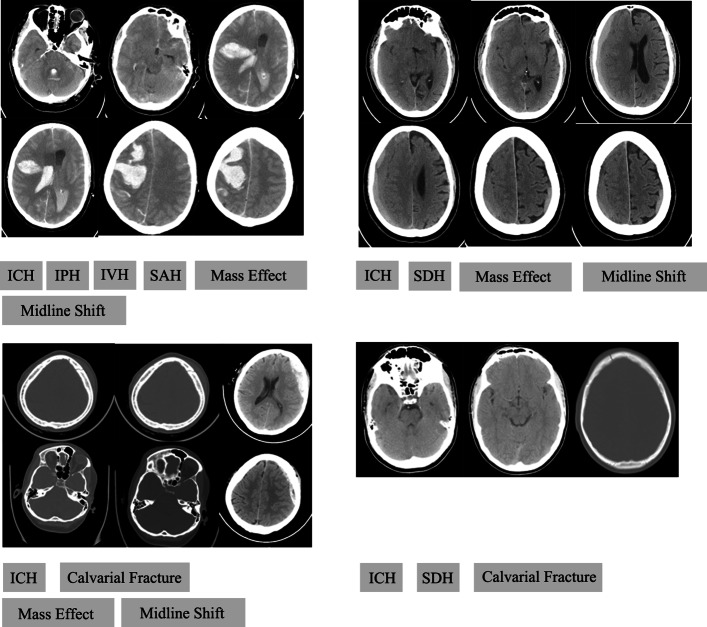
Example CT images from the dataset. We can see that a full-slice brain CT scan can reflect multiple diseases, and for an accurate diagnosis of the disease, multiple images are required. Therefore, we cannot only treat a single slice image. Learning the dependencies of the full-slice is very important. (ICH: Intracranial haemorrhage, IPH: Intraparenchymal, IVH: Intraventricular, SAH: Subarachnoid, SDH: Subdural)

In this study, experimental data are obtained from an open-source brain CT scan dataset called the CQ500 dataset [30]. This dataset contains 491 brain CT scans of patients and their reports labelled by three doctors. The datasets contain nine categories: intracranial haemorrhage (ICH), intraparenchymal (IPH), intraventricular haemorrhage (IVH), subdural haemorrhage (SDH), extradural haemorrhage (EDH), subarachnoid haemorrhage (SAH), calvarial fracture, mass effect, and midline shift. Negative samples are brain CT sequences that do not contain the above nine diseases. Example brain CT images can be seen in Fig. 6.

Causal relationships exist among the nine categories of brain diseases, e.g., ICH contains SDH, EDH, and SAH. In medicine, the mass effect is defined as the effect of a growing mass that causes secondary pathological effects when the mass pushes on or displaces the surrounding tissue. A possible cause of the mass effect is the haemorrhage blood or tumour. Furthermore, tumour or blood clot may constrict the brain and cause a midline shift. Therefore, the conventional multi-class or multi-label classification method may fail to explicitly exploit the label dependencies in a set of brain CT scans.

The original clinical radiology reports and consensus of three independent radiologists were considered as an evaluation metric for the CQ500 datasets. Because different radiologists have different judgments, we use evaluation metrics that most experts can satisfy. The three radiologists had corresponding experiences of 8, 12, and 20 years in cranial CT interpretation; however, they also had different judgments for the same scan. It is not difficult to observe that accurately diagnosing diseases through medical imaging is a difficult task for inexperienced doctors. Therefore, it is necessary to develop a system to assist diagnosis and treatment. The data statistics can be seen in Table 1.

**Table 1:** Data statistics

Disease	Number of patients
Intracranial haemorrhage	289
Intraparenchymal	145
Intraventricular haemorrhage	31
Subdural haemorrhage	49
Extradural haemorrhage	8
Subarachnoid haemorrhage	53
Calvarial fracture	71
Mass effect	102
Midline shift	74
Negative samples	187

We obtain 1194 complete sets of CT scans. Each patient has 1 to 8 scans. Because of the limited data, we use all types of brain CT scans together and only remove data that are not the brain CT scans. We divide the dataset into a train set containing 835 CT scan images, a validation set containing 180 CT scan images, and a test set containing 179 CT scan images. The objective of our classification is to cover all kinds of diseases comprehensively.

### Proposed model training

DenseNet, VGG, and ResNet are popular feature learning models. LSTM and GRU are two popular RNN networks whose performance are excellent when considering long-term dependencies. In the implementation of the experiments, three (two) dominant variants of CNN (RNN), i.e., VGG, DenseNet, ResNet (GRU and LSTM) are used for the comparison study. For each image from a set of brain CT, the feature learning model VGG (DenseNet and ResNet) generates a 512 (1024 and 2048) dimension vector. The total parameters of our model depend on the last layers of the output of the CNN and the RNN. The image extracted by VGG16 and VGG19 has the same output, i.e., 512 parameters. This is the reason that the 16-layer and 19-layer VGG networks have the same number of parameters if the RNN part is the same. The results (as shown in Table 2) demonstrate that the combination VGG16+GRU has the best performance and save many parameters.

**Table 2:** Comparison of different CNN and RNN pair

Model	Parameters	Precision	Recall	F1
ResNet50-GRU	10,106,889	63.56%	47.11%	0.5411
ResNet50-LSTM	13,253,641	57.35%	49.16%	0.5259
**VGG16-GRU**	5,382,153	67.57%	**61.04**%	**0.6412**
VGG16-LSTM	6,956,041	58.13%	57.87%	0.5794
VGG19-GRU	5,382,153	58.61%	57.49%	0.5802
VGG19-LSTM	6,956,041	46.89%	65.45%	0.5462
DenseNet121-GRU	6,957,065	60.93%	45.24%	0.5168
DenseNet121-LSTM	9,055,241	48.59%	47.62%	0.4883

**Fig. 7:**
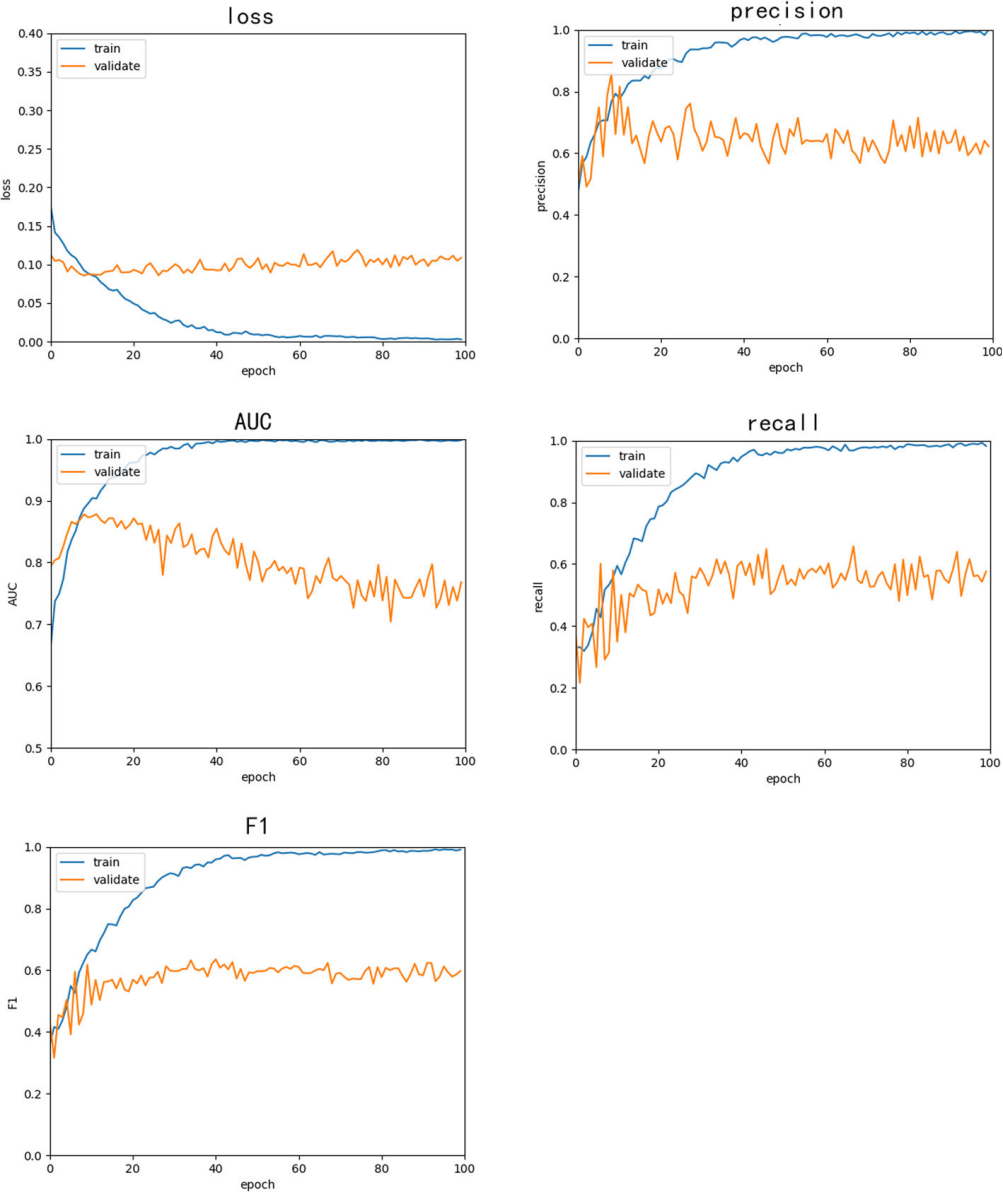
Training process of VGG16-GRU, which exhibits the best performance

We trained this model for 23 epochs. After 23 epochs, the performance of the model will not be better due to over-fitting, and the loss in the validation set will increase. The AUC will continue to decrease while training. The results of the training process are illustrated in Fig. 7. We use it to show the variation tendency of the performances (Precision, Recall, F1, AUC, Loss) with the increase of the epochs while the model training. Consequently, we terminate training after 23 epochs to avoid the over-fitting and obtain a better performance.

### Comparative experiment with 3-D models

The 3-dimensional convolutional networks (3-D ConvNets) can effectively learn spatiotemporal feature. Some studies, e.g., in references [12–15], attempt to utilize 3-D network in the field of brain CT classification. However, no research has shown that using 3-D models for multi-label brain image classification. We modified the VGG model to make it a 3-D model. We use the 3D-VGG and the classic C3D model [31] to do a multi-label classification comparison experiment. We modified two model’s last layer to output nine probabilities. We use the sigmoid function as the last layer’s activation function. It is the same as the model we proposed, we use MSE as our loss function to measures the average of the squares of the errors between the true labels and the predictions of the nine disease’s probabilities.

Since the 3-D model is too computationally intensive, it is impossible to consider hundreds of images at the same time as our model. We selected 32 slices from a set of *n* brain CT images (a full-slice) in steps of *n*/32. For training reason, we reshape the image into (112,112) pixels. Each set of data is a (32,112,112,3) tensor. We cannot remove too much data, because it will lose too much information. The batch size we set is 8. The result is shown in Table 3. These performances are worse than our model, and even if we greatly reduce the amount of data, the calculation of 3-D models is still much larger than our model. So our model is more suitable for this task.

**Table 3:** Comparison of different 3-D model and our model

Model	Parameters	Precision	Recall	F1
3D-VGG	94,512,329	49.37%	38.72%	0.4340
C3D	22,220,681	49.58%	44.89%	0.4712

### Model application for binary classification problem

**Fig. 8:**
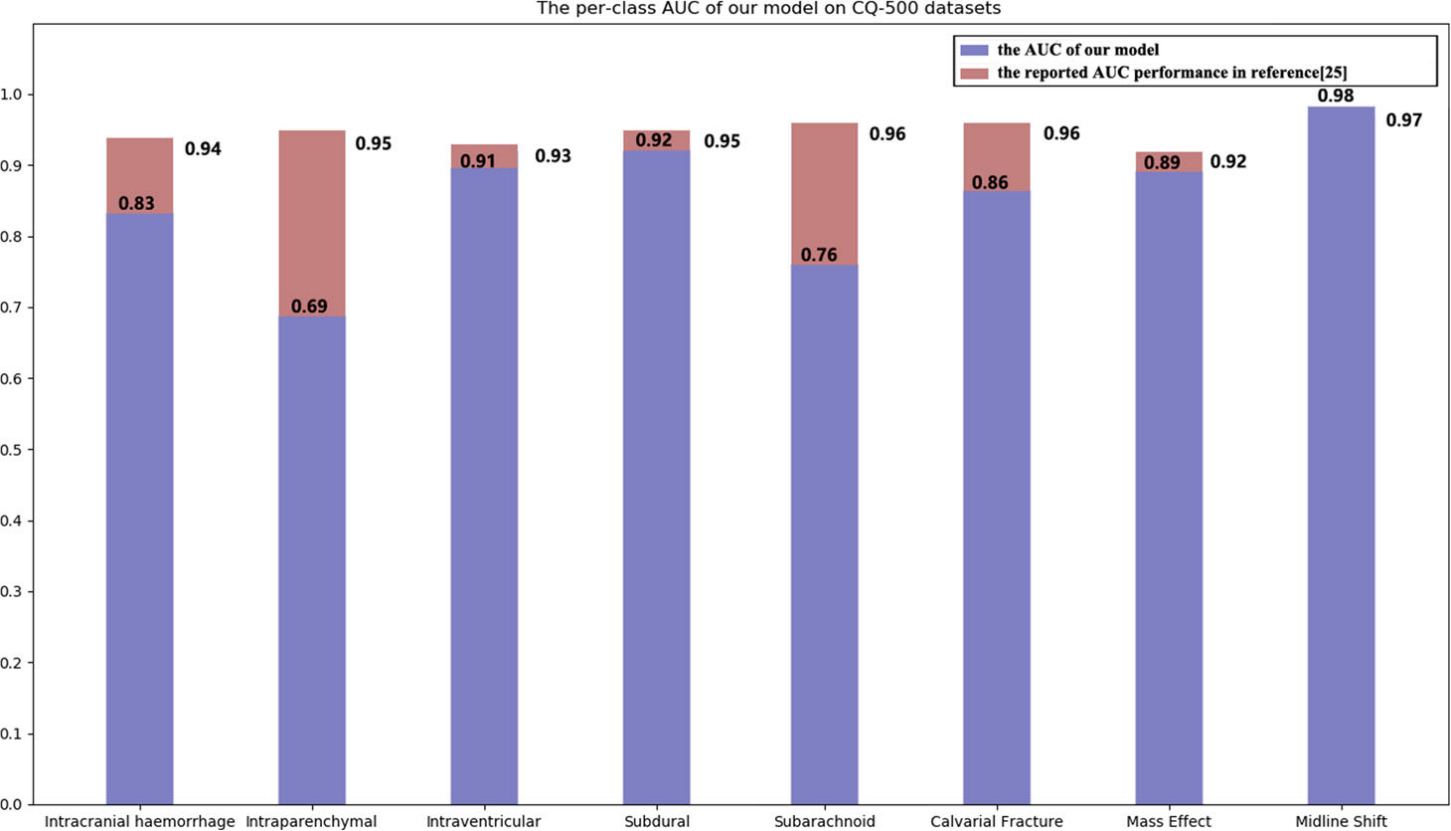
AUCs of the binary classifiers of our model and the original dataset model. There are only eight patients who have extradural haemorrhage; therefore, this binary classifier was not built

Our approach using a full set of slices directly for the multi-label predictive model building is different from the work reported in reference [30] focusing on single image-based binary classification. It needs the manual labelling of the haemorrhages in each selected image and cannot handle the dependency relationships among multiple slices. To make the experimental results comparable, we implemented a binary classifier version of our proposed model by mapping a multi-hot vector to the number 0 or 1 to indicate whether the image with the disease or not. The experimental results on the CQ500 dataset are illustrated in Fig. 8. The average AUC of our binary classifier version of our proposed model is 0.855. Note that, the experiments reported in this paper is conducted on the CQ500 dataset (i.e., the used training, validation, and test sets are all part of it), which contains 491 patients’ scans and is the released dataset in [30] for its performance testing. As reported in reference [30], its model is built from the training dataset with 313,318 patients’ scans. Consequently, the train and test datasets we used in this paper are different from [30]. From this viewpoint, it is not reasonable to compare the performance figures directly. If we have to give a reason for that the AUC of our proposed model is lower than the reported performance in [30], it might lie in the fact that the training dataset in this paper is much smaller than the one used in reference [30]. The results in Fig. 8 demonstrated that we use a very tiny dataset to obtain a great performance.

## Results

The precision, recall, and F1 score of the generated labels are employed as evaluation metrics. Precision is the number of correctly annotated labels divided by the number of generated labels, and recall is the number of correctly annotated labels divided by the number of ground-truth labels. The precision of our multi-label classification model is 67.57%, its recall score is 61.04%, and its F1 score is 0.6412. For binary classifiers version of our proposed model, the AUCs are 0.83, 0.69, 0.91, 0.92, 0.76, 0.86, 0.89, and 0.98 for ICH, IPH, IVH, SDH, SAH, calvarial fracture, mass effect, midline shift, respectively. Only eight patients have EDH; therefore, we did not construct a binary classifier for this disease.

## Discussions

In this study, we developed and investigated a new model that can automatically classify brain CT images into multiple categories at the same time. We adopted the multi-class classification into a multi-label classification problem. Our model does not require all slices in a set of brain CT scans to be labelled. This is one of the advantages of this work. However, its precision and recall are lower than models proposed in other research. The reason is that our model only contains the labels that full-slice brain CT images can reflect whereas other studies label every image in a set of scans. Consequently, they have more image information. Similar to how doctors diagnose brain diseases with a set of CT images, computer algorithms should also identify diseases with a set of CT images. We trained two 3-D models(3D-VGG, C3D) in the same data distribution. We modified two models and data for training. The result shows that the 3-D model is not suitable for this multi-label classification task because of the huge amount of calculation. We also trained our model using a binary classification problem and compared its results with those given in reference [30]. The experimental results prove that our performance is not considerably worse than other methods. However, we have only considered a small dataset, if more datasets are used, the algorithm will exhibit better performance. Besides, this method also saves the time spent on the label image.

We performed several experiments for different models. Table 2 presents that VGG performs better than other methods when the RNN parts are consistent, and VGG16 performs better than VGG19. The reason, that VGG is more suitable for this task than other models, is perhaps because VGG has a shallower convolutional layer. VGG16 has only 16 layers, however, other networks have a greater number of layers, such as DenseNet121 has 121 layers and ResNet50 has 50 layers. Even VGG16 and VGG19, which have nearly the same network architecture, exhibit different performance. In the same epoch, the F1 score of VGG16 is higher than that of VGG19. In the medical imaging domain, medical images only exhibit a subtle change when people are suffering certain kinds of diseases, unlike complex transformations that occur in natural images. Consequently, this could be a reason for the degradation in performance with an increase in the number of layers. The usage of AI techniques to recognize medical images and assistance in the diagnosis of the disease shows promising potential. In the future, we will attempt image augmentation on sequential images and increase their interpretability to better assist doctors in diagnosis.

## Conclusions

Brain CT scans are a common and useful tool and are used to provides accurate information on brain injuries, such scans can rapidly reveal internal injuries and help save lives. However, it is difficult to accurately identify diseases through brain CT scans, and even highly experienced doctors are prone to misdiagnosis. Acute brain diseases are life-threatening conditions that require rapid detection and treatment. Consequently, it is necessary to use deep learning methods to assist in the diagnosis. One set of brain CT scans can reflect multiple diseases. It is unreliable to use a single image in a brain CT scan for diagnosis, which is well investigated in conventional methods. Conventional methods ignore the causal relationship between brain diseases and the dependence between slices. Moreover, labelling a set of brain CT images one by one takes a considerable amount of time.

In this study, we proposed a model that learns the features of sequence images and their slice dependencies for multi-label classification in a full-slice brain CT scan. We classified brain CT scans into nine categories, and the F1 score of the model is 0.6412. The results presented in this work demonstrate that deep learning algorithms can be used to automatically detect the type of brain disease. This technology may have a potential application for clinical use and can improve healthcare delivery through the detection of a variety of acute diseases.

## Abbreviations

CT: Computerised tomography
SDLM: Slice dependencies learning model
AUCs: Areas under the receiver operating characteristic curves
OCT: Optical coherence tomography
AD: Alzheimer’s disease
CNN: Convolutional neural network
RNN: Recurrent neural network
GRU: Gated recurrent unit
SVM: Support vector machine
MR: Magnetic resonance
MCI: Mild cognitive impairment
HCP: Hypotheses-CNN-pooling
seq2seq: Sequence to sequence
PCA: Principal component analysis
LDA: Linear discriminant analysis
GPU: Graphics processing units
ResNet: Residual neural network
DenseNet: Densely connected convolutional network
ReLU: Rectified linear unit
LSTM: Long short-term memory
MSE: Mean squared error
ICH: Intracranial haemorrhage
IPH: Intraparenchymal
IVH: Intraventricular haemorrhage
SDH: Subdural haemorrhage
EDH: Extradural haemorrhage
SAH: Subarachnoid haemorrhage
